# Altholactone Inhibits NF-κB and STAT3 Activation and Induces Reactive Oxygen Species-Mediated Apoptosis in Prostate Cancer DU145 Cells

**DOI:** 10.3390/molecules22020240

**Published:** 2017-02-07

**Authors:** Chunwa Jiang, Muqaddas Masood, Azhar Rasul, Wei Wei, Ya Wang, Muhammad Ali, Muhammad Mustaqeem, Jiang Li, Xiaomeng Li

**Affiliations:** 1 The Key Laboratory of Molecular Epigenetics of MOE, Institute of Genetics and Cytology, Northeast Normal University, Changchun 130024, China; jiangcw833@nenu.edu.cn (C.J.); miss.nagra@yahoo.com (M.M.); azcheena37@hotmail.com (Y.W.); 2 Faculty of Science and Technology, Government College University Faisalabad (GCUF), Faisalabad 38000, Pakistan; drazharrasul@gmail.com (A.R.); alisam007@hotmail.com (M.A.); 3 Dental Hospital, Jilin University, Changchun 130021, China; drasharrasul@gmail.com; 4 Department of Chemistry, University of Sargodha, Sub-campus Bhakkar 30000, Pakistan; mustaqeem@uos.edu.pk; 5 Chemical Biology Research Group, RIKEN Center for Sustainable Resource Science, Chemical Biology Department, Wako, Saitama 351-0198, Japan

**Keywords:** altholactone, natural compound, prostate cancer, NF-κB, STAT3

## Abstract

Altholactone, a natural compound isolated from *Goniothalamus* spp., has demonstrated anti-inflammatory and anticancer activities, but its molecular mechanisms are still not fully defined. Nuclear factor kappa B (NF-κB) and signal transducer and activator of transcription 3 (STAT3) play pivotal roles in the cell survival of many human tumors. The objective of this study was to elucidate the mechanism of action of altholactone against prostate cancer DU145 cells and to evaluate whether its effects are mediated by inhibition of NF-κB and STAT3 activity. Altholactone inhibited proliferation of DU145 cells and induced cell cycle arrest in S phase and triggered apoptosis. Reporter assays revealed that altholactone repressed p65- and TNF-α-enhanced NF-κB transcriptional activity and also inhibited both constitutive and IL-6-induced transcriptional activity of STAT3. Consistent with this, altholactone down-regulated phosphorylation of STAT3 and moreover, decreased constitutively active mutant of STAT3 (STAT3C)-induced transcriptional activity. Altholactone treatment also results in down-regulation of STAT3 target genes such as survivin, and Bcl-2 followed by up regulation of pro-apoptotic Bax protein. However, pre-treatment with the antioxidant N-acetylcysteine (NAC) significantly inhibited the activation of Bax and prevented down-regulation of STAT3 target genes. Collectively, our findings suggest that altholactone induces DU145 cells death through inhibition of NF-κB and STAT3 activity.

## 1. Introduction

Prostate cancer is considered as one of the most common malignancies of men in the Western world [1] and the rate of occurrence has strikingly increased in the last two decades in Asian countries [2,3]. Importantly, the molecular mechanisms of prostate cancer, its development and progression are complicated phenomena because of the involvement of multiple factors, such as tumor suppressor genes, oncogenes, growth factors, signal transduction molecules, adhesion molecules, and angiogenesis [4]. Signal transducer and activator of transcription (STAT) proteins are involved in number of intracellular signals, in activation of immunity, apoptosis, and many other responses. The STAT proteins activation depends on the signaling of cytokines and growth factors [5]. STAT proteins are identified originally as key components of cytokine signaling pathways, are a family of latent cytoplasmic transcription factors those act as downstream of Janus-activated kinase or Src kinase activation and mediate intracellular signaling from a wide variety of cytokines, growth factors, and hormones [5,6]. Constitutive activation of STAT3 and its other family members has been detected in a variety of human tumor specimens and cancer cell lines, including prostate cancer [6], so there is a need for time to develop novel, improved therapeutic approaches and new therapeutic agents to cure prostate cancer. Anti-inflammatory agents that modulate inflammatory signaling pathways and cell proliferation may represent rational and pragmatic strategy to prevent or treat prostate cancers.

**Figure 1:**
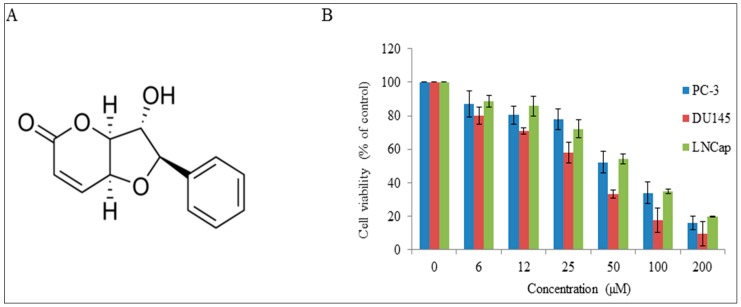
Chemical structure of altholactone and its effects on cell viability: (**A**) Chemical structure; (**B**) Altholactone inhibited the cell growth and induced cell death in prostate cancer cells. LNCaP, PC-3 and DU-145 cells were treated with the indicated doses of altholactone for 48 h and cell viability was measured by MTT assays. Data are expressed as mean ± SD (*n* = 3).

Natural products have received increasing attention as promising potential alternatives for preventing the progression of prostate cancer [7,8]. Among natural products, we and others have reported that sesquiterpene lactones demonstrate potential anticancer activities in different types of human cancers [9,10,11]. Altholactone is one of such agents that can inhibit cancer progression. Altholactone (Figure 1A), a natural tetrahydrofurano-2-pyrone, isolated from *Goniothalamus* spp., which belongs to the styryl-lactone family, has been reported to display anticancer activities in human colorectal cancer (HCRC) cells through caspases-dependent and independent apoptotic pathways [12], in the cervical carcinoma HeLa cell line by decreasing Bcl-2 and increasing p53 expression [13] and in leukemia HL-60 cells by induction of apoptosis via oxidative stress [14]. We previously reported that altholactone inhibited cell growth and induced apoptosis in human bladder cancer T24 cells by causing mitochondrial membrane potential imbalance followed by MAPK-p38 activation and suppression of the Akt pathway [15]. However, the details of the mechanism of action of altholactone remain unclear.

To date there are no reports of chemo-therapeutic effects of altholactone on human prostate cancer. Therefore, investigations have now been done for the first time to demonstrate the anti-proliferative potential of altholactone against human prostate cancer cells and then to delineate it’s underlying mechanisms of action. In this study, we revealed, by using DU145 cells as model, that altholactone inhibits transcriptional activity and phosphorylation levels of STAT3 in a dose-dependent manner. Further we present evidence that altholactone results in induction of reactive oxygen species (ROS) generation in prostate cancer DU145 cells, followed by activation of Bax and suppression of STAT3 target gene products, including Bcl2, and survivin.

## 2. Results and Discussion

### 2.1. Altholactone-Induced Cell Growth Inhibition in Prostate Cancer Cells

Natural plant products are an excellent potential source of novel anticancer agents. Over 70% of anticancer drugs developed in the last 30 years either are natural product-derived compounds from animals, plants and microorganisms [16]. The current study has been performed after random screening of Nature-derived drugs formerly selected from our own repositories. We choose compounds those were representative of specific classes of natural products we had previously reported [10,11,17,18]. The aim of this screening was to identify compounds that target ROS metabolism in cancer. Recently, we reported that altholactone induced ROS-mediated apoptosis in bladder cancer cells [15]. Here, we extend those previous studies to examine the cytotoxic potential of altholactone on prostate cancer cells. MTT assays were performed on two androgen-independent human prostate cancer cell lines (PC-3 and DU145) and an androgen-dependent cell line (LNCaP) to assess the dose-dependent cytotoxicity of the compound. Drug concentration, altholactone, and cell viability work inversely, as cell viability decreases in DU145 cells expressing constitutively active STAT3 as the drug concentration increases, with an IC_50_ (concentration to achieve 50% of cell growth) value of 38.5 µM. However drug exerted the lesser effect on PC-3 and LNCaP cells as comparedto the DU145 cells (Figure 1B). To support our previous results [15] that altholactone induces cytotoxic effects by targeting the ROS metabolism, pretreatment of DU145 with NAC (5 mM, a specific ROS inhibitor) was performed. The results showed that 5 mM NAC diminished the effect of alhtolactone on DU145 cells and support the notion that alhtolactone induces cytotoxic effects by targeting the ROS metabolism (data not shown). These findings are the parallel with the previous studies in which alhtolactone has been reported as a growth inhibitor of different cancer cell lines such as bladder cancer T24 cells [15], cervical carcinoma HeLa cells [13] and human colorectal cancer cells [12].

### 2.2. Effect of Altholactone on Induction of DU145 Cell Cycle

**Figure 2:**
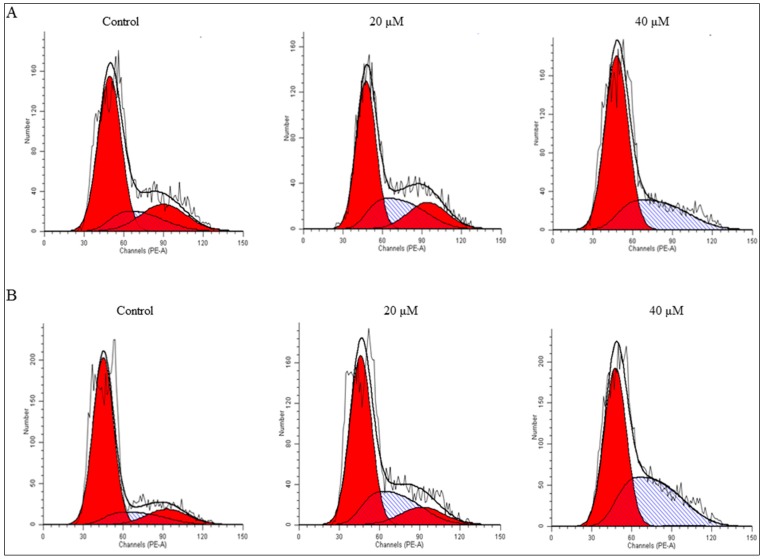
Flow cytometry analysis of cell cycle phase distribution in DU145 cells: (**A**) DU145 cells treated with 20 and 40 µM of altholactone for 24 h; (**B**) DU145 cells treated with 20 and 40 µM of altholactone for 48 h. Data are representative of three independent experiments (*n* = 3).

Recent reports related to cell cycle regulation have disclosed that cell cycle progression is tightly controlled by various checkpoints in normal cells and alterations in these checkpoints of cell cycle progression may lead to aberrant cell proliferation and development of cancer [19]. Cancer cells frequently acquire defects in the checkpoints and these results in cell cycle deregulation, which leads to unrestrained cell proliferation. Pharmacological corrections of these checkpoints followed by proper progression of cell cycle can be an effective strategy to control the growth and proliferation of cancer cells [19,20]. In order to evaluate the whether the antiproliferative effect of altholactone is due to inhibition of cell cycle progression or induction of apoptosis, next, we analyzed the effects of altholactone on cell cycle progression in DU145 cells. Flow cytometric data revealed that altholactone arrested the cell cycle at the S phase in a time- and dose-dependent manner. The accumulation of cells at S phase was coupled with a decreased percentage of cells in the G0/G1 and G2/M phases (Figure 2). These results clearly demonstrate that altholactone triggered cell cycle arrest in DU145 cells at the S phase, therefore, altholactone-induced cell death may results from cell cycle arrest. Our investigation also reveals that the altholactone-induced cell cycle arrest of DU145 cells appears to be one of the mechanisms of altholactone’s cytotoxic activity. Many contemporary studies have documented that numerous natural cytotoxic agents display potential anti-proliferative effects by arresting cell division at certain checkpoints of the cell cycle [11,21].

### 2.3. Effect of Altholactone on Induction of DU145 Cell Apoptosis

**Figure 3:**
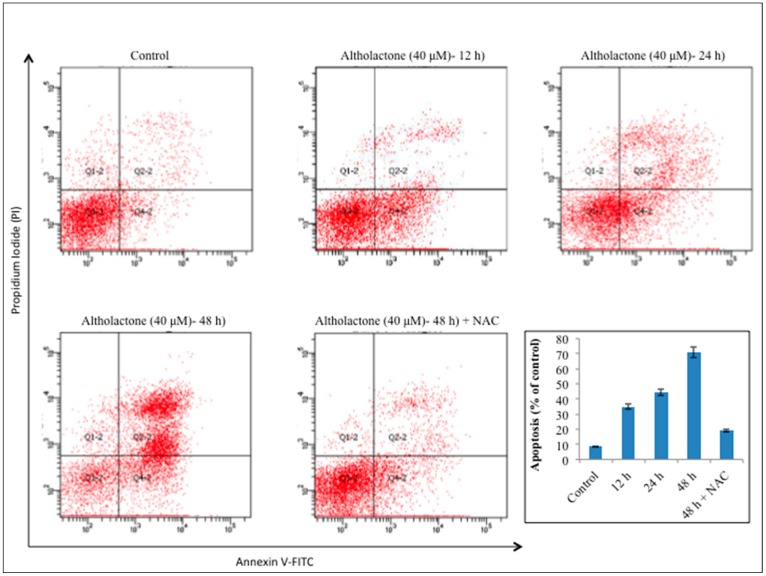
Apoptosis induced by altholactone in DU145 cells. The prostate cancer DU145 cells were treated with 40 µM of altholactone (AL) for 12 and 24 h or in the presence or absence of NAC for 48 h. Then cells were stained with FITC-conjugated Annexin V and PI for flow cytometric analysis. The flow cytometry profile represents Annexin V-FITC staining in *x* axis and PI in *y* axis. Data are representative of three independent experiments and are expressed as mean ± SD (*n* = 3).

In addition to the cell cycle arrest, another mode of cell death by natural compound is apoptosis, a programmed cell death which is characterized by morphological changes such as chromatin condensation and nuclear fragmentation [22,23]. Accumulated data from a number of recent studies [10,11,17,18,24] has proposed that a range of natural anticancer agents demonstrate anti-apoptotic potential to induce death in cancerous cells. To explore whether the inhibitory effect of altholactone on DU145 prostate cancer cells was associated with induction of apoptosis or not, DU145 cells were incubated with altholactone (40 µM) in a time dependent manner and the percentages of apoptotic/necrotic cells were measured by flow-cytometric analysis by using Annexin V-FITC and PI staining. Treated DU145 cells had a higher ratio of apoptosis as compared to the control cells. We observed that after 24 h or 48 h treatment DU145 cells show significantly increased percentages of both early and late apoptosis whereas after 12 h treatment DU145 cells show a significant increase only in early apoptosis as compared with the control group. Our results thus showed that altholactone induces apoptosis in a time-dependent manner. The flow cytometric analysis results showed that the rates of apoptosis were 32.72% ± 2.26%, 42.98% ± 3.14%, and 66.57% ± 2.96% for 12, 24, and 48 h, respectively, as compared to the 6.85% ± 2.89% in control cells (Figure 3).

Cancer cells interfere with the programmed cell death mechanism and these results in uncontrolled cell growth. Many reports have revealed that several anticancer drugs elevate the level of ROS in various types of cancer cells and these results in the activation of the programmed cell death mechanism [9,11,25]. To define the role of altholactone as ROS inducer in DU145 cells, cells were pretreated with ROS scavenger, NAC (5 mM) for 2 h, and then incubated with altholactone (40 µM) for an incubation time of 48 h. NAC pretreatment of the cells noticeably diminished the apoptotic effect of altholactone as compared to the cells which did not get NAC pre-treatment. Our findings indicate that induction of apoptosis in DU145 cells under altholactone treatment is ROS-dependent (Figure 3). Consequently, these outcomes suggest that altholactone treatment of DU145 cells results in elevated levels of intracellular ROS, which are followed by apoptotic cell death. These findings agree with our observed cell survival data and are also compatible with a previously reported study [15].

### 2.4. Altholactone Promoted ROS Generation and Inhibited NF-κB Activity in DU145 Cells

Reactive oxygen species (ROS) are natural byproduct of normal oxygen metabolism and perform important roles in cell signaling and homeostasis [26]. However in the onset, ROS is the product of mitochondrial malfunctioning and metabolic intensification, and there may be ways to selectively regulate the ROS levels in cancer cells [27]. As we reported earlier, alhtolactone induces ROS generation in bladder cancer cells [15]. Therefore, in this study, we assumed that alhtolactone may also have potential to induce ROS in DU145 cells and these results in alhtolactone-induced apoptosis. To confirm our assumption, intracellular ROS levels were measured by using the ROS-detecting fluorescence dye 2,7-dichlorofluorescein diacetate (DCF-DA). The level of ROS was significantly increased in a time-dependent manner after treating the cells with altholactone (40 µM) for 6, 12, and 24 h. We further confirmed our results by using NAC, a specific ROS inhibitor, whereby pretreatment with ROS scavenger results diminishes the effect of alhtolactone on treated DU145 cells.

**Figure 4:**
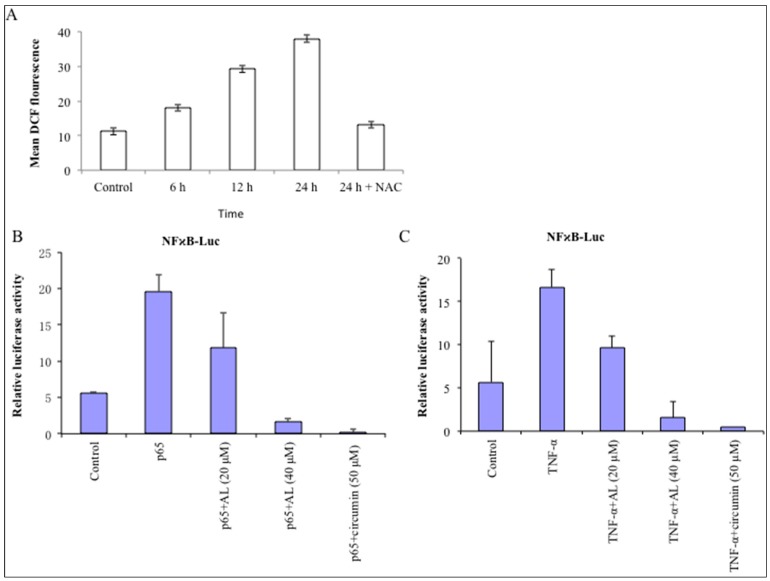
Altholactone promoted ROS generation and inhibited p65- and TNF-α-induced NF-κB transcriptional activity: (**A**) DU145 cells were treated with 40 µM altholactone (AL) for 6 and 12 h or in the presence or absence of 5 mM NAC for 24 h. Data are expressed as Mean ± SD (*n* = 3); (**B**) The effect of altholactone (AL) on the NF-κB transcriptional activity. DU145 cells were transiently co-transfected with p65 expression plasmid and NF-κB-Luciferase plasmid. At 16 h after transfection, cells were treated with the indicated concentrations of altholactone for 8 h and then harvested for luciferase and β-galactosidase assays; (**C**) the effect of altholactone on TNF-α-enhanced transcriptional activity of NF-κB. After 16 h of transfection, cells were treated with the indicated doses of altholactone (AL) for 8 h, and then prior to harvest for luciferase and β-galactosidase assays, cells were treated with TNF-α for stimulation. Curcumin, a NF-κB inhibitor, was used as positive control in this study. Data are expressed as mean ± SD (*n* = 3).

As shown in Figure 4, the ratio of DCF-positive cells, treated with 40 µM of altholactone for 6, 12, and 24 h was significantly higher (18.06 ± 2.32, 29.24 ± 3.54 and 37.96 ± 3.55 vs. 11.23 ± 2.42 in control group). These findings demonstrated that altholactone results in ROS production in DU145 cells (Figure 4A). Chemotherapeutic natural agents than can cause oxidative stress are supposed to be lethal for cancer cells because of their ability to control biological processes by induction of cell cycle arrest, DNA repair, and also by generation of apoptosis [15].

In order to further delineate the underlying molecular mechanism of altholactone induced ROS-mediated apoptosis in DU145 cells, we examined the effects of altholactone on the transcriptional activity of various transcription factors such as NF-κB, STAT3, and AP1 that all play pivotal roles in cancer progression. NF-κB is a transcriptional factor that activates cell survival and proliferation signals while it’s over activation may result in numerous types of cancer [28]. However, inhibition of NF-κB induced signaling can be a promising appropriate strategy to cure cancer and many other diseases. On the other hand, to date many chemotherapeutic natural compounds have been reported to induce apoptosis by inhibition of NF-κB signaling in various cancer cell lines [25,29]. It was previously reported that altholactone exhibits NF-κB based anti-inflammatory activity [30], and in the present study we found that altholactone inhibited p65- and TNF-α-enhanced NF-κB transcriptional activity in DU145 cells (Figure 4B,C). Altholactone also slightly repressed AP-1 transcriptional activity (data not shown). Curcumin, a NF-κB inhibitor, was used as positive control in this study.

### 2.5. Altholactone Inhibited Constitutive, IL6- and STAT3C-Induced STAT3 Transcriptional Activity and STAT3 Phosphorylation in DU145 Cells

**Figure 5:**
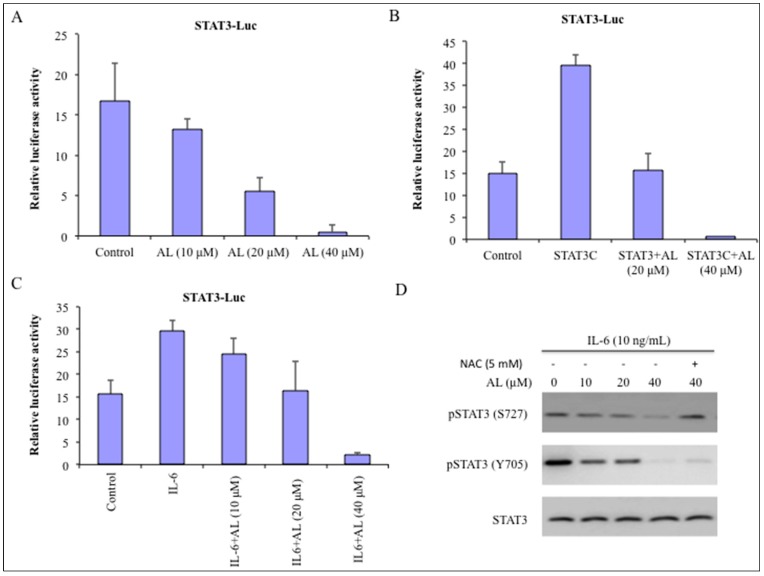
Altholactone inhibited constitutive, IL6- and STAT3C-induced STAT3 transcriptional activity and STAT3 phosphorylation in DU145 cells: (**A**) the effect of altholactone on the STAT3 transcriptional activity. DU145 cells were transiently transfected with M67-Luciferase plasmid. After 16 h of transfection, cells were treated with the indicated concentrations of altholactone for 8 h and then harvested for luciferase and β-galactosidase assays; (**B**) The effect of altholactone on IL-6-enhanced transcriptional activity of STAT3. At 16 h after transfection, cells were treated with the indicated doses of altholactone for 8 h and then prior to harvest for luciferase and β-galactosidase assays, cells were treated with IL-6 for stimulation; (**C**) DU145 cells were co-transfected with M67-Luc construct and the expression plasmid for constitutive STAT3 (STAT3C). At 16 h after transfection, cells were treated with altholactone for 8 h then harvested for luciferase and β-galactosidase assays. Data are the means of three independent experiments, each performed in duplicate; (**D**) Altholactone inhibited phosphorylation of STAT3 at Tyr705 and Ser727. Serum-starved cells for 24 h followed by altholactone treatment (10, 20 and 40 µM) for 8 h prior to stimulation with IL-6. To examine the role of ROS, cells were pre-treated with NAC (5 mM) for 2 h followed by altholactone (40 µM) treatment. Cells were harvested for western blotting using antibodies against pSTAT3 (Y705), pSTAT3 (S727), and STAT3. Data are the representative of three independent experiments.

Signal transducer and activator of transcription-3 (STAT3) is an oncogenic transcriptional factor, which is constitutively activated in more than 50% of primary tumor and tumor-derived cell lines [31,32]. Studies have shown that oxidative stress results in glutathionylation of STAT3 followed by concomitant inhibition of its phosphorylation [17]. In other words, NF-κB is an activator of STAT3 signaling. Because altholactone results in reduced expression of NF-κB activity in DU145 cells moreover DU145 cells which constitutively express active STAT3 were more sensitive to altholactone, therefore, we were curious to find whether altholactone could also inhibit STAT3 activation. In order to examine the effect of altholactone on the transcriptional activity of STAT3, we carried out transient transfection and luciferase assays using DU145 cells. Altholactone significantly repressed the constitutive and IL-6 stimulated STAT3 transcriptional activity in DU145 cells (Figure 5A,C).

To define the molecular mechanisms of inhibitory effect of altholactone, next we analyzed the expression of IL-6 stimulated phosphorylation at Tyr705 (pTyr705 STAT3) and Ser727 (pSTAT3 S727) in DU145 cells. As shown in Figure 5D, altholactone treatment decreased the expression of pTyr705, and pSTAT3 S727 in a dose-dependent manner while total STAT3 expression remained unchanged. To further validate whether altholactone could modulate STAT3 activity independently of upstream signaling, cells were transiently transfected with a constitutively active form of STAT3 (STAT3C) that mimics the activity of phosphorylated STAT3 [6,21]. Interestingly, altholactone significantly inhibited the STAT3C-stimulated transcriptional activity (Figure 5B). These results give the notion that altholactone may inhibit the constitutive STAT3 transcriptional activity by binding to the SH2 domain of STAT3. Further studies are required to examine binding mode of altholactone with STAT3.

To comprehend the role of elevated level of ROS in the phosphorylation of STAT3 inhibition, DU145 cells were pre-treated with NAC (5 mM) for 2 h followed by altholactone (40 µM) incubation for the indicated time interval. Proteins were extracted, prepared and immuno-blotted with antibodies against pSTAT3 (Y705), pSTAT3 (S727), and STAT3. As shown in Figure 5D, altholactone treatment resulted in down-regulation of STAT3 phosphorylation but pretreatment with NAC prevented altholactone-induced inactivation of STAT3 phosphorylation. This data clearly demonstrates that altholactone treatment causes release of ROS that, in turn, causes inhibition of STAT3 phosphorylation. However the precise means by which ROS release leads to STAT3 phosphorylation inhibition is yet not known.

### 2.6. Effect of Alotholactone on STAT3 Gene Products and on Apoptosis-Related Proteins

Tyrosine phosphorylation (Tyr705) of STAT3 is associated with increased expression of genes involved in cell proliferation process such as Bcl-2 and survivin [21]. Many reports have established that survivin, a new member of the inhibitor of apoptosis (IAP) family, is accompanied with both cancer progression and drug resistance. However distraction of STAT3 signaling pathway results in decreased expression of anti-apoptotic proteins and leads to tumor cell apoptosis [33]. 

**Figure 6:**
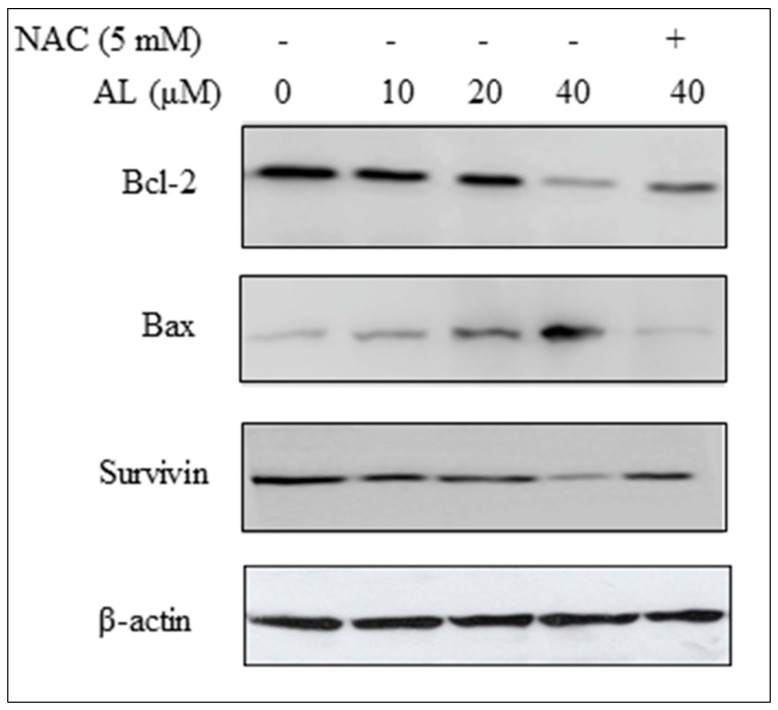
Western blot analyses of STAT3 downstream target genes and pro-apoptotic Bax protein. DU145 cells were treated with altholactone (10, 20 and 40 µM), and then harvested for western blotting. Equal amounts of lysate protein were subjected to gel electrophoresis. β-Actin was used as loading control. Data are representative of three independent experiments with similar results.

The interplay between anti-apoptotic (Bcl-2) and pro-apoptotic (Bax) proteins of the Bcl-2 family have been well documented. To further explore whether altholactone can modulate the transcription of STAT3 downstream targets products and pro-apoptotic Bax protein, western blot analyses of STAT3 downstream target genes and Bax protein was performed. DU145 cells were treated with various concentration of altholactone for the indicated time periods and then harvested for preparation of protein lysates. The data demonstrated that altholactone significantly down regulate the protein levels of target gene’s products and up-regulate expression of Bax protein while pretreatment with NAC prevented the effect of altholactone (Figure 6). These findings demonstrate that altholactone decreased expression of proteins of STAT3 target gene transcription.

## 3. Materials and Methods 

### 3.1. Chemical, Plasmid Constructsand Other Reagents

Altholactone was purchased from the BioPha Co., Ltd. of Pharmaceutical and Biological Products (Kunming, China) and dissolved in 100% DMSO (20 mM for stock solution). Reporter constructs (NF-κB-Luc, and M67-Luc) were a kind gift from Professor Dr. David A. Frank (Department of Medicine, Harvard Medical School and Brigham and Women’s Hospital, Boston, MA, USA) [34] and reporter constructs (STAT3 Luc and AP-1-Luc) were from Professor Stephen Yarwood (University of Glasgow, Glasgow, UK) [35]. The expression plasmid for a constitutively active form of STAT3 (STAT3C) was a kind gifts from Dr. Feng Li (Department of Pharmacology, Yong Loo Lin School of Medicine, National University of Singapore, Singapore) and p65 expression plasmid was obtained from Professor Soo Young Lee (Division of Molecular Life Sciences and Center for Cell Signaling Research, Ewha Womans University, Seoul, Korea) [36]. Cell culture medium reagents and MTT [3′-(4,5-dimethylthiazol-2-yl)-2,5-diphenyltetrazolium bromide], propidium iodide (PI), and dimethyl sulfoxide (DMSO) were purchased from Sigma (St. Louis, MO, USA). Fetal bovine serum (FBS) was purchased from the Hangzhou Sijiqing Biological Engineering Materials Co., Ltd. (Hangzhou, China). Annexin V-FITC apoptosis detection and reactive species detection kits were purchased from Beyotime Institute of Biotechnology (Shanghai, China). Ponceau and cell lyses buffer for western blots and IP were purchased from Bio SS Beijing (Beijing, China). IL-6 was purchased from BD Biosciences (San Jose, CA, USA).

### 3.2. Cell Culture

The two androgen-independent human prostate cancer cell lines (PC-3 and DU145) and an androgen-dependent cell line (LNCaP) were allowed to proliferate in DMEM nutrients mixture which was supplemented with 10 %FBS and antibiotics, incubated at 37 °C in humidified atmosphere (5% CO_2_ and 95 %air). Cells were seeded in 10 cm culture dish and allowed to grow to approximately 70% confluence before harvesting the cells for experiments.

### 3.3. Cell Proliferation Assay

The cytotoxic effects of the altholactone on two androgen-independent human prostate cancer cell lines (PC-3 and DU145) and an androgen-dependent cell line (LNCaP) were determined by using an MTT assay [29]. Briefly, cells were seeded in 96-well plates at a density of 4000 cells per well and allowed to grow overnight. In each well cells were incubated with 200 µL of complete culture medium containing 0, 6, 12, 25, 50, 100, and 200 µM of altholactone. After 48 h of incubation with the drug, cell viability was determined by adding 10 µL of MTT (5 mg/mL in phosphate buffered saline) solution to each well and allowed to incubate for 4 h. After removal of the medium, 150 µL DMSO was added to each well and shaken carefully. The absorbance was read at a wavelength of 570 nm in a plate reader (ELX 800, BIO-TEK Instruments Inc., Winooski, VT, USA). The growth curve was plotted against mean values which were calculated by using the following equation:

I% = [A570 (control) − A570 (treated)]/A570 (control) × 100(1)

### 3.4. Flow Cytometric Determination of Cell Cyle Propagation

The effects of the altholactone on cell cycle progression of DU145 cells were determined as we described previously [37].

### 3.5. Flow Cytometric Determination of Apoptosis

Apoptosis rate of DU145 cells was examined by flow cytometry by using annexin V-FITC/PI staining [11]. Briefly, DU145 cells were cultured in 6-well plates and allowed to attach overnight. Cells were incubated with 40 µM concentration of altholactone for 12 and 24 h. Then cells were harvested, rinsed off and resuspended in PBS buffer. Apoptotic cell death was measured by using double staining annexin V-FITC and PI using the annexin V-FITC apoptosis detection kit (Beyo time Institute of Biotechnology) according to the manufacturer’s protocol. Flow cytometric analysis was done within 30 min after staining. Data acquisition and analysis were performed by flow cytometry using the Cell Quest software.

### 3.6. Flow Cytometric Determination of Reactive Oxygen Species (ROS) in DU145 Cells

In order to investigate the intracellular changes in ROS generation, DU145 cells were stained with 2′,7′-dichlorofluoresceindiacetate (DCFH-DA) as described earlier [11]. The fluorescent dye DCFH-DA is permeable to cell membranes and intracellular esterases converted it into the cell membrane impermeable non-fluorescent compound DCFH. Oxidation of DCFH by reactive oxygen species produces highly fluorescent DCF. The fluorescence intensity of DCF inside the cells is directly proportional according to the amount of peroxide produced. Briefly, DU145 cells were treated 40 µM altholactone for 6, 12 and 24 h. After treatment, cells were further incubated with 10 µM of DCFH-DA at 37 °C for 30 min. Consequently, cells were harvested, rinsed off with PBS, re-suspended in PBS, filtered with 300 apertures and analyzed for 2′,7′-dichlorofluorescein (DCF) fluorescence by flow cytometry (Epics XL, Beckman Coulter, Miami, FL, USA).

### 3.7. Trasient Transfection and Luciferase Assays

To reveal the effect of altholactone on transcriptional activities of NF-κB and STAT3, luciferase assays were performed as we reported previously [3]. Briefly, transient transfections were performed using Lipofectamine 2000 (Invitrogen, Shanghai, China) by following the manufacturer’s protocol. Cells were seeded into 48-well plates for 16 h and transfected or co transfected with each of reporter plasmids 100 ng or with 25 ng of expression plasmid in the presence of Renilla luciferase control pREP7 vector 25 ng, and then treated with altholactone ranging from 0 to 40 μM for 8 h. Firefly luciferase activities were calculated by using the dual-luciferase reporter assay system (Promega, Madison, WI, USA) and the ratio of firefly luciferase activity to Renilla luciferase activity was measured as relative luciferase activity.

### 3.8. Western Blotting

To divulge the apoptotic effect of altholactone on DU145 cells, western blot analysis was performed for apoptotic related proteins as previously described [38]. Briefly, DU145 cells were incubated with 40 µM concentration of altholactone for indicated time periods. After that cells were washed with PBS and trypsinized, collected in centrifuge tube (1.5 mL) and then again washed with PBS. The cell pellets were resuspended in lysis buffer followed by lysed on ice for 30 min. After that centrifuge for 15 min, the supernatant were collected in another 1.5 mL Appendrof and the protein concentration was measured by using NanoDrop 1000 spectrophotometer (Thermo Scientific, Waltham, MA, USA). The protein lysates were separated by using technique of electrophoresis on 8%–12% SDS-polyacrylamide gel and transferred to a PVDF membrane (Amersham Biosciences, Piscataway, NJ, USA). The membranes were soaked in blocking buffer (5% skimmed milk) for 1 h. To probe, membranes were incubated with relevant antibodies STAT3, phospho-STAT3 Tyr705 (pSTAT3 Y705), phospho-STAT3 Ser727 (pSTAT3 S727), Survivin, and Bcl-2 (Cell Signaling, Beverly, MA, USA); Bax (Santa Cruz Biotechnology, Santa Cruz, CA, USA), and β-actin (Sigma-Aldrich) at 4 °C for overnight, followed by appropriate HRP conjugated secondary antibodies and ECL detection.

### 3.9. Statistical Analysis of Data

Statistical analysis of data was done by using SPSS for Windows version 15.0 (Chicago, IL, USA). Results are analyzed by comparison between different groups. Student’s *t-*test was used to verify the statistical significance of the differences between different experimental groups and control group. Statistically significant values was defined as *p* < 0.05 value. All the experiments were repeated for three times to conform the results and were presented as mean ± standard deviation (S.D).

## 4. Conclusions

In conclusion, we have revealed that altholactone exhibits cytotoxic effects against DU145 prostate cancer cells. To the best of our knowledge, we report here for the first time that the cytotoxic activity of altholactone is mediated at least in part by inhibition of NF-κB and STAT3 transcriptional activities and subsequent repression of transcription of STAT3 target genes. Therefore, our findings support that altholactone could represent a novel chemotherapeutic natural agent or lead scaffold against cancers that harbor constitutively active STAT3. Since the concentration of altholactone required to inhibit proliferation is rather high, more potent analogs of altholactone need to be developed for practical use in cancer treatment. Our data together with previous reports demonstrated that altholactone exerted its cytotoxic activities through multiple signaling pathways. Zhao and Li [15] have reported that altholactone exerts its in vitro anticancer action by Akt inhibition. Hence, considering current and previous findings, there is the possibility that altholactone may also act via inhibition of the PI3K-Akt-mTOR pathway that plays a key role in oncogenesis [39,40] and with possibility that altholactone promoted p53 expression, as previously has been reported [13,41], could mediate altholactone-induced anticancer action. Further in vivo or preclinical studies are needed to elucidate the anticancer potential of altholactone in combination or adjuvant therapies for the treatment of cancers.
